# The Measurement and Preliminary Application of the Concept of Family Centrality

**DOI:** 10.3389/fpsyg.2022.911292

**Published:** 2022-06-10

**Authors:** Xinran Wan, Wei Wang, Shengnan Wang, Geyan Shan, Yongxin Li

**Affiliations:** ^1^Institute of Psychology and Behavior, Henan University, Kaifeng, China; ^2^School of Education, Nanyang Normal University, Nanyang, China

**Keywords:** employee wellbeing, family involvement, work-family relationship, work centrality, family centrality

## Abstract

Family centrality refers to value judgment regarding the relative importance of family in an individual’s life. In contrast to bidirectional research in the field of work-family relationships, much work had been done about the work centrality, whereas few works of research discussed family centrality as an independent concept. Thus, the present study systematically discussed the concept of family centrality in Chinese culture and the preliminary validation of its measurement through two cross-sectional studies. In study 1, questionnaires were distributed to two sub-samples through convenient sampling; one included 185 participants (mean age of 35.51 ± 10.30) and other included 189 participants (mean age of 31.39 ± 6.82). In study 2, through convenient sampling, questionnaires were distributed to 351 participants with a mean age of 35.15 (*SD* = 9.44) years. Results of Study 1 supported that the Family Centrality Questionnaire (FCQ) has a single-factor structure with good reliability and validity. Additionally, family centrality and work centrality are two independent concepts that can be distinguished on conceptional and applicational levels. Results of Study 2 showed that family centrality had an indirect effect on life wellbeing through life involvement (β = 0.073, 95% CI [0.032, 0128]), and work centrality had an indirect effect on work wellbeing through work involvement (β = 0.089, 95% CI [0.046, 0.142]). Further, family centrality had a spillover effect on work wellbeing through work involvement (β = −0.079, 95% CI [−0.125, −0.42]), and work centrality has a spillover effect on life wellbeing through family involvement (β = −0.053, 95% CI [−0.095, −0.22]). Overall, the results showed that the FCQ can be used as a scientific measurement for future research.

## Introduction

Work and family are two important domains of an individual’s life, and the relationship between them is also a significant theme in the field of industrial and organizational psychology (Li and Huang, 2007). In the field of work-family relations, most concepts are bidirection, for instance, work-family conflict and family work conflict; work-family enrichment and family work enrichment. However, empirical studies are mainly focused on work centrality, emphasizing work and the workplace, thereby paying relatively less attention to family (e.g., Carr et al., 2008). Especially in China, the Confucian culture emphasis more importance about the families; however, previous studies (e.g., Carr et al., 2008; Ranihusna and Wulansari, 2015) neither systematically explained the concept of family centrality nor further tested the reliability, validity, and discrimination of the revised questionnaire. Therefore, this study aims to develop a measurement of family centrality.

The discussion of work centrality originated from the protestant ethic theory, “calling,” thus, the concept of vocation is the core of Protestantism. The Meaning of Work International Research Team (MOW) (Patrickson, 1988) first put forward the concept of “work centrality,” which is a central variable that defines the meaning of working. Carr et al. (2008) indicated that work centrality should be compared with other fields to reflect its significance. Therefore, they defined the work-family centrality as a value judgment about the relative importance of work to personal life. For instance, Carr et al. (2008) have treated low scores of work centrality as equivalent to being family centered (Carr et al., 2008; Ranihusna and Wulansari, 2015). Additionally, the work-family centrality questionnaire developed by Carr et al. (2008) has been the most widely used measure to assess work centrality. Although prior studies (e.g., Marks, 1977; Cinamon and Rich, 2002a,b) showed that work and family centralities are not mutually exclusive, and Bagger and Li (2012) further indicated the possibility of the co-existence of family centrality and work centrality, they have revised the work centrality questionnaire (Paullay et al., 1994) to measure family centrality. However, they have neither systematically explained the concept of family centrality nor further tested the reliability, validity, and discrimination of the revised questionnaire.

According to data from the World Values Survey (WVS) and the Chinese General Social Survey, people worldwide are emphasizing the significance of family. For instance, the importance of work centrality has decreased significantly among young people (Twenge and Kasser, 2013; Lukeš et al., 2019). Additionally, data from the WVS shows that the importance of work in China is declining year by year, with the percentage of people reporting that work is important falling from 63.4% in the first survey in 1990 to 38% in 2013. Moreover, the number of people who believe that family is important has increased from 62% in 1990 to 85.7% in 2013. According to the 2012 Chinese Workplace Balance Index Research Report, more than half of the post-1990s generation prioritized family over work. This result and development trend is not only consistent with the traditional Chinese value of “familism” (家族主义) based on the five ethics (五伦), but it also demonstrates the practical value of conducting the research on family centrality.

Family has played an important role in the traditional Chinese context. The basic characteristic of Confucian (儒家) culture, which has been respected by the Chinese since ancient times, is Confucian “Ren Lun”(人伦). “Lun” refers to interpersonal relationships. The “Tian Lun” (天伦), which refers to relationships with blood relatives and in-laws, is considered to be the basis of “Ren Lun.” “Familism” is an important feature of traditional Chinese values, where the “family” usually refers to interpersonal relations, which leads to the idea and practice of familism that stands for always putting the family first (Yang, 2005). As the primary component of Chinese social orientation, “familism” is a complex native cultural phenomenon with special connotations and functions (Lu, 2008). Jin et al. (2009) has systematically explored the structure and characteristics of Chinese values, and indicated that “human emotion” and “family standard” are important components of Chinese values, which further proves the important position of the family in the Chinese value system. Although traditional family values are becoming increasingly diversified, there are no significant inter-generational differences in family standards (Xu, 2013). Family cohesion facilitates strong resilience and adaptability, and its profound cultural accumulation is far beyond that of modernization (Wang, 2016). Above all, regardless of the rapid development of social economy and violent changes in social structure, the “family” has always played an important role in the Chinese self-concept.

Concerning the importance of the family, empirical studies showed that family centrality may have important effects on the individual or company. For instance, previous studies have shown that work centrality was positively related to work attitude and satisfaction, and the effects of work centrality on satisfaction could be moderated by national culture (e.g., Lu et al., 2019). Yuen et al. (2018) showed that family centrality was positively related with adolescent adjustment and could buffer the negative effect of dysfunctional family. Additionally, Bagger et al. (2008) indicate that family centrality could moderate the relationship between family work conflict and turnover intention. Moreover, Bagger and Li (2012) further found a three-way interaction, indicating that when work centrality was low, family centrality could moderate the relationship between family work conflict and job satisfaction. Although few empirical studies have been conducted in China, the connotation of family centrality has been reflected in relevant studies. The academic concepts of “familism” and “family standard” are similar to that of family centrality. In ancient China, the family was regarded as the core of personal life, and the interests of the individual always gave way to family interests. The “family standard” and “human emotion” related values of contemporary Chinese individuals also contain family oriented attitudes related to the importance given by an individual to the family. This is consistent with the connotations of family centrality.

Therefore, according to the connotation of family centrality reflected in Chinese values and in reference to Carr et al.’s. (2008) statement on work centrality, in the present study, family centrality is defined as an individual’s value judgment of the relative importance of family in personal life. It is an important component of Chinese values as well as the core of family values, which are influenced by a person’s socioeconomic background and personal characteristics. Family centered individuals consider their family role an important part of their self-concept, unlike “familism,” which takes the individual as an appendage of the family and weakens one’s subjective consciousness. Family centrality is a normative belief and behavioral tendency related to the importance of the family in an individual’s subjective consciousness. Rather than placing individuals and families in complete opposition, it represents a behavioral orientation in which family related content dominates the psychological process of the individual.

Above all, in view of the importance of the family in the Chinese context; nevertheless, the existing research lacks measurement tools for family centrality. Therefore, this study aimed to explore the concept of family centrality, develop a measurement tool for family centrality, and then make a preliminary attempt to apply it to explore the impact of work centrality and family centrality on employees’ wellbeing.

## Study 1

In order to study family centrality as a separate concept, the first step should be the development of a measure with high reliability and validity. Therefore, the purpose of Study 1 was to develop the Family Centrality Questionnaire (FCQ) by translating and revising the Work-family Centrality Questionnaire of Carr et al. (2008). Furthermore, the conceptional independence of family centrality and work centrality and the measurement reliability and validity were examined.

### Method

#### Participants

##### Produce

In study 1, through the convenient sampling method, questionnaires were distributed to two sub-samples through convenient sampling; one included 185 participants (mean age of 35.51 ± 10.30) and other included 189 participants (mean age of 31.39 ± 6.82).

##### Subsample 1

Subsample 1 for study 1 comprised 185 employees with a mean age of 35.51 years (*SD* = 10.30). There were 99 females (55.0%) and 81 males (45.0%). Among them, 88 (49.2%) were unmarried, and 89 (50.8%) were married. The majority of the participants (82.0%) were ordinary employees.

##### Subsample 2

Subsample 2 comprised 189 employees with a mean age of 31.39 years (*SD* = 6.82). There were 87 females (47.0%) and 98 males (53.0%). Among them, 87 (46.3%) were unmarried, and 101 (53.7%) were married. The majority of the participants (68.6%) were ordinary employees.

#### Measurement

##### Work Centrality

Work centrality was assessed using Carr et al.’s. (2008) Work-family Centrality Questionnaire (WCQ). The questionnaire contained five items, including “Work should be considered central to life rather than family.” These items were rated on a five-point Likert scale ranging from 1 (“completely inconsistent”) to 5 (“completely consistent”), with a higher score representing higher work centrality. In the present study, Cronbach’s α was.93.

##### Family Centrality

Family centrality was assessed by modifying the work centrality items in the WCQ to refer to the respondent’s family. This procedure has been used successfully in previous work-family conflict research (Frone and Rice, 1987) and family involvement research (Frone et al., 1992). Specifically, the FCQ contained five items (e.g., “Family should be considered more central to life rather than work”). These items were rated on a five-point Likert scale ranging from 1 (“completely inconsistent”) to 5 (“completely consistent”), with a higher score representing higher work centrality. In the present study, Cronbach’s α was.95.

##### Relative Centrality of the Family

To test the validity of the FCQ, relative family centrality and relative family importance were selected as calibration measures. We used the Relative Centrality of Family Scale (Patrickson, 1988) to measure relative family centrality. The MOW group divided work centrality into absolute work centrality and relative work centrality and used the method of measuring relative work centrality to measure the participants’ relative family centrality. Compared to the FCQ, the comparison range and scoring method of the Relative Centrality of Family Scale is different; however, we chose to perform the calibration as they all reflect the degree of family centrality. Participants were asked to divide a total of 100 points among the following domains to indicate the relative centrality in life at present: (a) leisure, (b) community, (c) work, (d) religion, and (e) family. The number of points assigned to the family indicates its relative centrality in the respondent’s life.

##### Relative Importance of the Family

The Relative Family Importance Questionnaire is a part of the WVS, which aims to determine what people value most in life. It is consistent with the connotation of family centrality; therefore, it was considered fit for calibration. Participants were asked to rate the importance of six main areas of life (family, friends, leisure, politics, work, and religion) using a four-point Likert scale ranging from 1 (“not important at all”) to 4 (“very important”). According to the formula used by Lu et al. (2019), the relative importance of the family is calculated by dividing the importance score of the family domain by the importance score of all the six domains combined.

##### Work-Family Conflict

Work-family conflict was assessed using the Work-Family Conflict Scale (Netemeyer et al., 1996). The scale comprises two factors, each including five items: work-family conflict (e.g., “Your work needs affect your family life”) and family work conflict (e.g., “Your or your family’s needs affect your work-related activities”). Items were rated using a seven-point Likert scale, ranging from 1 (“completely inconsistent”) to 7 (“completely consistent”), with a higher score representing a higher conflict. In the present study, Cronbach’s α was.78.

##### Work-Family Enrichment

Work-family enrichment was assessed using the Work-Family Enrichment Scale (Tang et al., 2009). The scale, developed in the context of the Chinese culture, has satisfactory reliability and validity and is more suitable for localization applications (Tang et al., 2009). The scale comprises two factors, each including seven items: work-family enrichment (e.g., “Work helps me to listen and understand different points of view, and helps me to perform better with my family”) and family work enrichment (e.g., “Being with my family helps me to be more caring, considerate and better able to deal with problems at work”). Items are rated on a five-point Likert scale, ranging from 1 (“completely inconsistent”) to 5 (“completely consistent”), with a higher score representing higher enrichment. In the present study, Cronbach’s α was.89.

#### Data Analysis

Data were analyzed using SPSS 23.0 and AMOS 23.0.

For subsample 1, first, exploratory factor analysis (EFA; estimated by the maximum likelihood method) was conducted on five items of the FCQ to test the scale structure. Then, EFA was conducted on 10 items of the FCQ and WCQ to test conceptual independence.

For subsample 2, first, confirmatory factor analysis (CFA; estimated by maximum likelihood method) was conducted to examine the structural validity of the FCQ by AMOS 23.0. Second, CFA was conducted to examine the independence of family centrality and work centrality by AMOS 23.0. Third, the correlation analysis was conducted to examine the external validity of the FCQ. Additionally, to further verify the validity, one-way ANOVA was conducted to examine whether the FCQ and WCQ could distinguish individuals with different work-family relationships (conflict and enrichment).

### Results

Exploratory factor analysis was conducted on five items of the FCQ. The result of eigenvalue recommends a single factor structure; when the number of factors was 1, the eigenvalue was above 1. A single factor could explain 69.52% of the variance. The result of EFA (maximum likelihood method and Promax rotation) is shown in Table 1. The factor loading of all five items was greater than 0.63, and the Cronbach’s α value was 0.888. Additionally, the result of CFA is also shown in Table 1. The result showed an acceptable goodness of fit (χ^2^ = 14.143, *df* = 5, *p* < 0.00, CFI = 0.990, GFI = 0.973, RMSEA = 0.099, SRMR = 0.015), and the Cronbach’s α was.946, which preliminary supported the structural validity of the FCQ.

**TABLE 1:** Results of the exploratory factor analysis (EFA) and confirmatory factor analysis (CFA) (Family Centrality Questionnaire).

Item	Factor loadings (EFA)	Factor loadings (CFA)
Q1. In my view, an individual’s personal life goals should be family oriented rather than work-oriented.	0.634	0.811
Q2. The major satisfaction in my life comes from my family rather than work.	0.828	0.845
Q3. The most important things that happen to me involve my family rather than work.	0.872	0.944
Q4. Family should be considered central to life rather than work.	0.834	0.918
Q5. Overall. I consider family to be more central to my existence than work.	0.761	0.859

The results of the correlation analysis were presented in Table 2. Family centrality was positively correlated with the relative centrality of family (*r* = 0.443, *p* < 0.01) and family importance (*r* = 0.211, *p* < 0.01). In general, these results showed the FCQ had a good external validity.

**TABLE 2:** Results of the correlation analysis among study 1 variables.

	①	②	③
① Family centrality	1.00		
② Relative Centrality of family	0.211**	1.00	
③ Relative importance of family	0.443**	0.460**	1.00

**p< 0.01.

Exploratory factor analysis was conducted on 10 items of the FCQ and WCQ. The result of the eigenvalue recommends a 2-factor structure; when the number of factors was 2, the eigenvalue was above 1. The two factors could explain 65.21% of the variance. The results of EFA (maximum likelihood method and Promax rotation) is shown in Table 3. When conducting CFA, in view of the fact that the contents of the FCQ and WCQ are consistent, a multi-trait multi-method model was created to verify the independence of the concepts of family centrality and work centrality. Ten items of the FCQ and WCQ were loaded on their respective constructs, and the residuals of items with the same content were allowed to be correlated. The results showed an acceptable goodness of fit (χ^2^ = 70.862, *df* = 29, *p* < 0.000, CFI = 0.975, GFI = 0.934, RMSEA = 0.088, SRMR = 0.042). The factor loadings of the 10 items are shown in Table 3.

**TABLE 3:** Results of the EFA and CFA (10 items).

Item	Loadings (EFA) (EFA)		Loadings (CFA)	
	FC	WC	FC	WC
Q1. In my view, an individual’s personal life goals should be family oriented rather than work-oriented.	0.630		0.811	
Q2. The major satisfaction in my life comes from my family rather than work.	0.831		0.847	
Q3. The most important things that happen to me involve my family rather than work.	0.871		0.943	
Q4. Family should be considered central to life rather than work.	0.833		0.918	
Q5. Overall, I consider family to be more central to my existence than work.	0.764		0.862	
Q6. In my view, an individual’s personal life goals should be work-oriented rather than family-oriented.		0.792		0.666
Q7. The major satisfaction in my life comes from my work rather than family.		0.827		0.858
Q8. The most important things that happen to me involve my work rather than family.		0.825		0.912
Q9. Work should be considered central to life rather than family.		0.857		0.899
Q10. Overall, I consider work to be more central to my existence than family.		0.795		0.859

*FC, family centrality; WC, work centrality.*

The participants in subsample 2 were divided into four groups using SPSS cluster analysis according to their scores on the FCQ and WCQ: low-family centered and high-work centered group (L-FC and H-WC), low-family centered and low-work centered group (L-FC and L-WC), high-family centered and high-work centered group (H-FC and H-WC), and high-family centered and low work-centered group (H-FC and L-WC). Results of the one-way ANOVA suggests that our grouping had a significant effect on the total score of work-family conflict (*F* = 12.487, *p* < 0.001), and the subscale of work to family conflict (*F* = 14.306, *p* < 0.001) and family to work conflict (*F* = 4.239, *p* < 0.01); the total score of work-family enrichment (*F* = 3.641, *p* < 0.01), and the subscale of family to work enrichment (*F* = 4.819, *p* < 0.01). According to the results of the post-test, the degree of conflict experienced by the L-FC and L-WC group and the L-FC and H-WC group was significantly lower than that experienced by the H-FC and L-WC group and the H-FC and H-WC group. Similarly, the degree of enrichment experienced by the L-FC and L-WC group and the L-FC and H-WC group was significantly higher than that of the H-FC and L-WC group and the H-FC and H-WC group (Table 4).

**TABLE 4:** Result of one-way ANOVA.

Group	N	W-FC	F-WC	WFC	W-FE	F-WE	WFE
		*M* ± *SD*	*M* ± *SD*	*M* ± *SD*	*M* ± *SD*	*M* ± *SD*	*M* ± *SD*
L-FC and H-WC	35	11.54 ± 5.04	11.11 ± 4.86	22.66 ± 9.20	26.34 ± 6.10	26.43 ± 6.11	52.77 ± 11.70
L-FC and L-WC	43	13.49 ± 4.82	11.33 ± 4.39	24.81 ± 7.98	26.09 ± 5.65	27.14 ± 5.12	53.23 ± 10.15
H-FC and H-WC	57	16.05 ± 3.32	12.79 ± 3.77	28.84 ± 5.06	24.00 ± 4.33	24.79 ± 4.69	48.79 ± 8.09
H-FC and L-WC	54	17.57 ± 5.46	13.94 ± 4.64	31.52 ± 8.02	24.85 ± 5.46	23.28 ± 5.82	48.12 ± 8.41
*F*	-	14.306***	4.239**	12.487***	1.983	4.819**	3.641**
LSD	-	1, 2 < 3, 4	1, 2 < 4	1, 2 < 3, 4	-	1, 2 > 3, 4	1, 2 > 3, 4

**p < 0.01; ***p < 0.001. W-FC, work to family conflict; F-WC, family to work conflict; WFC, work family conflict; W-FE, work to family enrichment; F-WE, family to work enrichment; WFE, work-family enrichment.

## Study 2

Study 1 provided preliminary support that family centrality and work centrality are two separated concepts, and the FCQ showed good reliability and validity. Further, Study 2 aims to determine the effects of family centrality and work centrality on employee happiness as well as the spillover effect of role centrality in the field of work and family.

### Research Hypotheses

Role centrality, which represents an individual’s belief, attitude, and value orientation, is a generalized, stable, persistent, resistant, and dynamic belief system (Meglino and Ravlin, 1998). Work and family centrality, as important components of work and family values, are critical to understanding the meanings of work, life, and wellbeing (Kittel et al., 2019). Moreover, role centrality is a result of socialization (Paullay et al., 1994), and the life role is an important component of an individual’s self-concept; it emphasizes a kind of self-identity (Mannheim, 1975). Self-identity is positively correlated with life satisfaction and subjective wellbeing (Cui et al., 2015). Additionally, Jin et al. (2009) found a significant correlation between values and life satisfaction as well as self-concept and life satisfaction. Based on this, we propose hypothesis 1.

*Hypothesis 1a*: There is a positive relationship between family centrality and life wellbeing.

*Hypothesis 1b*: There is a positive relationship between work centrality and work wellbeing.

Additionally, according to the self-determinism theory, values determine inner needs, and satisfaction of psychological needs affects individuals’ wellbeing (Greguras and Diefendorff, 2009; Yen, 2012; Slemp and Vella-Brodrick, 2014). As part of the value system, role centrality determines internal needs, and employees’ expectations of work depend on their inner needs. Researchers have demonstrated that values affect subjective wellbeing through cognition (Oishi et al., 1999).

Work involvement and family involvement are two aspects of self-involvement. On the one hand, as a belief system of individuals, involvement is guided and restricted by their values; on the other hand, “involvement” itself is an element of individual wellbeing (Schaufeli et al., 2002), belonging to high-pleasure emotional experience with high emotional arousal (Bakker and Oerlemans, 2011). Involvement makes people more likely to succeed by inducing a positive emotional state and a greater sense of mastery, thereby satisfying their inner needs, and the satisfaction of psychological needs brings happiness (Zou et al., 2015).

Based on Rabinowitz et al.’s (1977) comprehensive theory of work involvement, personal characteristics, such as intrinsic motivation, work intention, and self-esteem, affect work involvement, which in turn affects employee wellbeing. Fan (2014) also proved that work involvement plays a partial mediating role in the relationship between job values and job satisfaction. However, there are few studies on family involvement. Nevertheless, as a belief proposed in parallel with work involvement, it is also influenced by family values and can further affect employee happiness. Thus, involvement plays an important role in the relationship between role centrality and wellbeing. We propose hypothesis 2 below.

*Hypothesis 2a*: The relationship between family centrality and life wellbeing could be mediated by family involvement.

*Hypothesis 2b*: The relationship between work centrality and work wellbeing could be mediated by work involvement.

According to the theory of spillover and compensation, individuals’ emotions, attitudes, behaviors, and skills at the workplace could spill over to the family domain and vice versa (Staines, 1980). These spills can be either positive or negative. Positive spillovers bring satisfaction and motivation; negative spillovers generally refer to the consumption of time and energy in one field, such that individuals are unable to take care of the other field. Compensation theory holds that work-family spillovers are negative when individuals have a higher level of involvement in the work domain, and the level of involvement in the family domain decreases (Staines, 1980). Further, in the studies of effects of work-family relationship on satisfaction, the results were inconsistent; this may attribute to a third moderation variable (Zhao and Li, 2007). Further, Wang et al. (2012) indicated that the inconsistent result may stem from the role differences.

According to the conservation of resource theory (COR; Hobfoll, 2001), with limited recourse, consuming time, energy, and cognitive resources in one field will lead to a scarcity of available resources in another field. Both work and family roles are important social roles for individuals, and the amount of energy and time they distribute to a role depends on the extent of the importance of work or family roles they viewed. A high level of psychological involvement in one role can make it more difficult to cope with the stresses associated with another role; it can also lead to mental distraction from the other role when physically trying to satisfy the demands of the second role (Greenhaus and Beutell, 1985).

However, the degree of role centrality may affect the permeability of work and family boundaries (Allen et al., 2014). Matthews et al. (2010) proposes that permeability is the transition from work to family, that is, the number of physical and cognitive changes from one field to another. Kossek et al. (2011) also propose cross-role interruption behavior, that is, the extent to which an individual allows interruption from one role to another. For example, high work-centered and low family centered individuals are less likely to become involved in the family, as they have high levels of work-to-family permeability and low levels of family to work permeability. Therefore, we propose hypothesis 3 as below.

*Hypothesis 3a*: The relationship between family centrality and work wellbeing would be mediated by work involvement.

*Hypothesis 3b*: The relationship between work centrality and life wellbeing would be mediated by family involvement.

**FIGURE 1:**
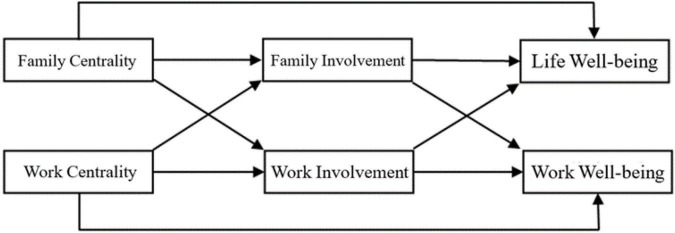
Model based on the hypotheses.

Based on the above hypotheses, we propose a two-path model of role centrality that influences employee wellbeing, as shown in Figure 1.

### Method

#### Participants

The sample comprised 351 employees with a mean age of 35.15 years (*SD* = 9.44). There were 187 females (54.7%) and 155 males (45.3%). Among them, 123 (35.0%) were unmarried, and 228 (65.0%) were married. The majority of the participants (74.9%) were grassroots employees.

#### Measurements

##### Family Centrality and Work Centrality

Same as study 1.

##### Work Involvement and Family Involvement

Work involvement was assessed using five items from Frone et al.’s. (1992) Work involvement Scale (e.g., “Most of my interests are centered around my job”). These items were rated on a six-point Likert scale ranging from 1 (“strongly disagree”) to 6 (“strongly agree”), with a higher score representing higher work involvement. In the present study, Cronbach’s α was.87. Family involvement was assessed with a parallel set of five items from Frone et al.’s. (1992) Family Involvement Scale (e.g., “Most of my interests are centered around my family”). The scoring method was the same as the work involvement scale. In the present study, Cronbach’s α value was.80.

##### Work Wellbeing and Life Wellbeing

Work wellbeing was assessed using six items from Zheng et al.’s (2015) Work Wellbeing Scale, a subscale of the Employee Wellbeing Scale (e.g., “Work is a meaningful experience for me”). These items were rated on a six-point Likert scale ranging from 1 (“strongly disagree”) to 6 (“strongly agree”), with a higher score representing higher work wellbeing. In the present study, Cronbach’s α was.87. Life wellbeing was assessed using six items from Zheng et al.’s (2015) Life Wellbeing Scale, a subscale of the EWB Scale (e.g., “Most of the time, I do feel real happiness”). The scoring method was the same as that of the work wellbeing scale. When testing this scale, we found that the item “If there is an afterlife, I will hardly change my present lifestyle” could not be loaded onto the factor of life wellbeing. The Cronbach’s α value was.86 after deleting this item.

#### Data Analysis

Before testing the hypotheses, common method variance was examined by controlling for the effects of the unmeasured latent method factor (Podsakoff et al., 2003) using AMOS 23.0. A correlation analysis was then conducted for a preliminary examination of the hypotheses using SPSS 23.0. Then, the mediation effects of role involvement and the spillover effect of role centrality were examined by constructing a structural equation model and bootstrap analyses using AMOS 23.0.

### Results

All the measures were evaluated using the same source. Before testing the hypotheses, the discriminant validity was examined by CFA. The measurement model—which allowed every item to load on its respective construct—was compared with two nested models. The measurement model comprised six factors: family centrality, work centrality, family involvement, work involvement, life wellbeing, and work wellbeing, and the model showed acceptable goodness of fit (χ^2^ = 881.54, *df* = 403, *p* < 0.01, CFI = 0.90, RMSEA = 0.06, SRMR = 0.07). The first nested model comprised three factors, which combined family centrality with work centrality, family involvement with work involvement, and life wellbeing with work wellbeing (χ^2^ = 2183.00, *df* = 415, *p* < 0.01, CFI = 0.62, RMSEA = 0.11, SRMR = 0.13). The second nested model comprised two factors, which combined family centrality, family involvement, and life wellbeing as one factor (χ^2^ = 3217.07, *df* = 417, *p* < 0.01, CFI = 0.40, RMSEA = 0.14, SRMR = 0.19) and work centrality, work involvement, and work wellbeing as one factor. The third factor combined all six factors into one (χ^2^ = 3952.16, *df* = 465, *p* < 0.01, CFI = 0.32, RMSEA = 0.15, SRMR = 0.25). The results showed that the nested model was significantly worse than the measurement model, which suggested the measurement used in this study showed good discriminant validity.

Then, the common method variance was examined by controlling for the effects of the unmeasured latent methods factor (Podsakoff et al., 2003). A CFA model was built, and each item was loaded on its respective construct (i.e., family centrality, work centrality, family involvement, work involvement, life wellbeing, and work wellbeing). Additionally, a common method variance factor was created, and all items were allowed to load on it. The latent factor did not correlate with other factors. The variance explained by the latent method factor was 4%, which is lower than the median of 25% shown in previous work (Williams et al., 1989). These results provide further evidence that the common method variance had little effect on the overall results of the present study.

Means, standard deviations, and correlations for the variables of study 2 are presented in Table 5. Family centrality was positively correlated with work centrality (*r* = 0.15, *p* < 0.01), family involvement (*r* = 0.32, *p* < 0.01), and negatively correlated with work involvement (*r* = –0.18, *p* < 0.01). Similarly, work centrality was positively correlated with work involvement (*r* = 0.21, *p* < 0.01) and negatively correlated with family involvement (*r* = −0.22, *p* < 0.01). Family involvement was positively correlated with life wellbeing (*r* = 0.18, *p* < 0.01), work involvement was positively correlated with work wellbeing (*r* = 0.36, *p* < 0.01), and life wellbeing was positively correlated with work wellbeing (*r* = 0.52, *p* < 0.01). However, there was no significant correlation between family centrality and life wellbeing (*r* = −0.018, *p* = 0.736) or between work centrality and work wellbeing (*r* = 0.077, *p* = 0.151). Generally, these results provided preliminary support for H2 and H3, while H1a and H1b were not supported.

**TABLE 5:** Means, standard deviations, and correlations among the variables of study 2.

Variable	*M*	*SD*	1	2	3	4	5	6	7	8	9	10	11	12
(1) Gender	1.55	0.499												
(2) Age	35.15	9.437	−0.161**											
(3) Working age	12.04	10.473	−0.180**	0.922**										
(4) Education level	2.75	0.765	−0.001	−0.259**	−0.302**									
(5) Position	1.28	0.502	−0.174**	0.227**	0.186**	0.192**								
(6) Married status	1.65	0.478	−0.010	−0.132*	−0.148**	−0.017	−0.048							
(7) Family centrality	15.10	3.700	−0.147**	−0.031	0.012	−0.111*	−0.140**	0.037	(0.796)					
(8) Work centrality	13.74	3.615	−0.079	−0.055	−0.029	−0.120*	0.046	0.080	0.153**	(0.832)				
(9) Family involvement	4.080	0.8013	−0.191**	0.106*	0.088	0.005	−0.019	−0.045	0.323**	−0.211**	(0.808)			
(10) Work involvement	3.693	0.8583	−0.135*	0.122*	0.120*	−0.023	0.128*	−0.109*	−0.183**	0.215**	0.079	(0.791)		
(11) Life Wellbeing	20.11	4.396	0.038	0.062	0.036	−0.043	0.175**	0.101	−0.018	−0.043	0.175**	0.149**	(0.862)	
(12) Work Wellbeing	23.82	5.293	−0.011	0.139**	0.100	−0.076	0.193**	0.096	−0.219**	0.077	−0.047	0.362**	0.519**	(0.886)

*p < 0.01; **p < 0.001.

Structural equation modeling was used to evaluate the model. Referring to previous research, marriage, gender, working years, and the position may potentially affect wellbeing (Wu et al., 2012). Because some of them were not correlated with wellbeing, controlling for too many variables would have decreased overall analytical power (Becker, 2005). Therefore, according to the results of correlation analyses, marital status and position were entered into the model as the control variables. The theoretical links between these variables and the experience of happiness are not the subject of this study; therefore, they are not discussed here. The hypothetical model showed a poor fit (χ^2^ = 137.35, *df* = 13, CFI = 0.63, GFI = 0.92, RMSEA = 0.165). The path coefficients of family centrality → life wellbeing (*p* = 0.545), work centrality → work wellbeing (*p* = 0.468), and family involvement → work wellbeing (*p* = 0.172) were not significant.

**FIGURE 2:**
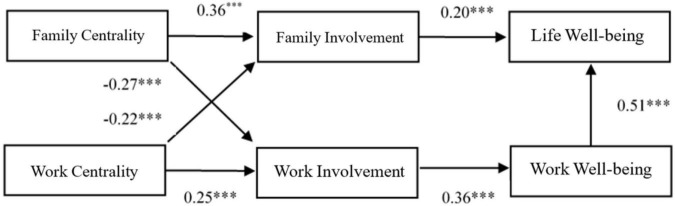
Path coefficient diagram. For clarity, the control variables and their paths were not marked. ***p < 0.001.

Considering the Chinese “family oriented” values, individuals may emphasize family more than work, and “work is for a better life” make work a certain “instrument” for the family. Additionally, the present study uses the life wellbeing questionnaire to measure employees’ family wellbeing, and although work is an important field of personal life, work wellbeing will have a strong effect on employees’ overall life wellbeing. According to the results of the previous correlation analysis, the two outcome variables, work wellbeing and life wellbeing, are significantly correlated. Thus, work wellbeing and life wellbeing may have important causal relationships. Combined with theory and practice, this study added a path from work happiness to life happiness, deleting the insignificant path, and obtained the final revised model. The result showed a good fit (χ^2^ = 34.84, *df* = 16, CFI = 0.94, GFI = 0.98, RMSEA = 0.058). The results of the final model are shown in Figure 2.

According to Figure 2, family centrality positively predicted family involvement (β = 0.36, *p* < 0.001), family involvement positively predicted life happiness (β = 0.20, *p* < 0.001), work centrality positively predicted work involvement (β = 0.25, *p* < 0.001), and work involvement positively predicted work happiness (β = 0.36, *p* < 0.001). These results provide preliminary support for Hypotheses 2a and 2b. Contrastingly, family centrality negatively predicted work involvement (β = −0.27, *p* < 0.001) and work centrality negatively predicted family involvement (β = −0.22, *p* < 0.001), providing preliminary support for hypotheses 3a and 3b. However, family involvement had no significant effect on work wellbeing, and work involvement had no significant effect on life wellbeing. Therefore, the spillover effect related to family centrality indirectly influencing work wellbeing through family involvement, and work centrality indirectly influencing life wellbeing through work involvement is untenable.

Further, the bias-corrected bootstrap was used to test the significance of the mediating effect. The non-parametric bootstrapping method (*n* = 2000) was used, with the 95% confidence interval calculated using the bias-corrected bootstrapping method. The results show that family involvement has a significant mediating effect on the relationship between family centrality and life wellbeing (β = 0.073, 95% CI [0.032, 0128]), and work involvement has a significant mediating effect on the relationship between work centrality and work wellbeing (β = 0.089, 95% CI [0.046, 0.142]), thus supporting hypotheses 2a and 2b (Table 6).

**TABLE 6:** Summary of estimates and bias-corrected bootstrapped 95% confidence intervals.

			Bias-corrected 95%CI		
Path	Estimate	SE	Lower	Upper	*p*
Family Centrality → Family Involvement → Life Wellbeing	0.073	0.024	0.032	0.128	0.001
Family Centrality → Work Involvement → Work Wellbeing	−0.079	0.021	−0.125	−0.042	0.001
Work Centrality → Work Involvement → Work Wellbeing	0.089	0.024	0.046	0.142	0.001
Work Centrality → Family Involvement → Life Wellbeing	−0.053	0.018	−0.095	−0.022	0.001
Family Centrality → Work Involvement → Work Wellbeing → Life Wellbeing	−0.040	0.012	−0.067	−0.021	0.001
Work Centrality → Work Involvement → Work Wellbeing → Life Wellbeing	0.045	0.013	0.023	0.075	0.001

In the test of the spillover effect, the spillover effect related to family centrality indirectly influencing work wellbeing through work involvement (β = −0.079, 95% CI [−0.125, −0.42]), and work centrality indirectly influencing life wellbeing through family involvement (β = −0.053, 95% CI [−0.095, −0.22]) were significant, thus supporting hypotheses 3a and 3b.

Additionally, after adding the path of work wellbeing to life wellbeing, the new chain mediating paths of work centrality to work involvement to work wellbeing to life wellbeing (β = 0.045, 95% CI [0.075, 0.001]) and family centrality to work involvement to work wellbeing to life wellbeing (β = 0.045, 95% CI [0.075, 0.001]) were generated. The results show that high role centrality has both a positive and negative effect on wellbeing.

## Discussion

In the research of work-family relation, research on work or workplace have attracted more attention than those on families (Amstad et al., 2011). However, the importance of family was also apparent; as noted by Payne (2020) family is the most relevant and central relation among all the relationships we have with others, both personal and professional familial relationships. Although some studies mentioned family centrality, few have examined family centrality as an independent variable. For instance, Carr et al. (2008) have treated low scores of work centrality as equivalent to being family centered (Carr et al., 2008; Ranihusna and Wulansari, 2015). Similarly, Xie et al. (2015) also used the term “work-family centrality,” defining work-family centrality as a value judgment about the relative importance of the work. However, both theoretical and empirical research has suggested that work and family centralities are not mutually exclusive (Marks, 1977; Cinamon and Rich, 2002a,b). Therefore, in this study, we developed a family centrality questionnaire, providing preliminary support for its reliability and validity. The results of Study 1 also provided new evidence that family centrality and work centrality are two different concepts.

To further explore the consequence of work centrality and family centrality, specifically, examine whether work centrality has specific effects on work domain variables and whether family centrality has specific effects on family domain variables. Results of Study 2 generally supported our hypotheses. In line with prior studies, work centrality was positively related to work involvement (Paullay et al., 1994), and work involvement was positively associated with work wellbeing (Knoop, 1995). Furthermore, work involvement plays a complete mediating role in the relationship between work centrality and work wellbeing. Similarly, the family domain found the same effect, that family involvement plays a complete mediating role in the relationship between family centrality and life wellbeing. At the same time, family centrality has a negative spillover effect on work wellbeing, and work centrality has a negative spillover effect on life wellbeing.

## Theoretical Implication

First, the current study refers to relevant research on work centrality, combined with the context of Chinese family values, systematically expound the concept of family centrality, which enriched our understanding of role centrality and Chinese family values. Additionally, family centrality and work centrality are two independent concepts, indicating that it is necessary to consider family centrality and work centrality in parallel in future studies on role values.

Second, the results of the difference test showed that the differences in work-family relationships (conflict and enrichment) mainly exist among individuals with different degrees of family centrality. This indicates that compared with work centrality, family centrality has a greater impact on Chinese people, further confirming the importance of focusing on family centrality in China. Furthermore, the higher the level of family centrality, the greater the work-family conflict and the lower the work-family enrichment. This may be due to the “priority” and “instrumentality” of work in relation to the family in a collectivistic culture. Compared with the equality of work and family in western society, work and family in the eastern collectivistic culture are asymmetrical, and work is more important than family (Hofstede, 2007; Zhang et al., 2009, 2011). In China, hard work is regarded as a traditional virtue, and working overtime is often seen as a sacrifice made for the family. Studies have also proved that Chinese individuals are more tolerant of work-related intrusions in their family lives, and employees are less concerned about work-family conflict (Bu and McKeen, 2000). Moreover, in a collectivistic culture, work for the family has a strong “instrumental” color, and individual development in a way is contributing to the family; therefore, an emphasis on work can also reflect an emphasis on the family. Wang et al. (2004) also noted that in a collectivistic culture, individuals define job success as an event that glorifies one’s family. The conflict between an individual’s high level of family centrality and the priority and instrumentality of work tend to make people with higher family centrality experience more work-family conflict and less work-family enrichment.

Third, the results of this study suggest that role involvement plays a complete mediating role in the relationship between role centrality and wellbeing. Consistent with previous studies, the effect of trait emotion on satisfaction is mediated by state effects (Weiss, 2002; Ilies and Judge, 2004). In the model of the formation mechanism of work wellbeing, personal characteristics do not directly affect work wellbeing but play a moderating role in the influencing process (Bowling et al., 2005).

Fourth, the results of this study demonstrate that role centrality has a negative spillover effect on the wellbeing of employees in another field, which supports the COR theory and spillover theory. There are three possible explanations for the negative effects of family centrality on work wellbeing. According to the COR theory, people with high family centrality devote more time and energy to the family; therefore, devote less time and energy to their work, leading to lower levels of work involvement. Low levels of work involvement mean less positive emotional states at work and lower returns, which further reinforces job unhappiness. Additionally, when employees are “forced” to work because of external pressure, it inevitably results in the occupation of family resources (time and energy), and individuals may feel that work “encroaches” on the family domain, resulting in dissatisfaction with their current job. Furthermore, if employees are voluntarily involved in work because of high rewards or community pressure, those with high family centrality may experience a sense of dissonance in their attitudes and behaviors; this feeling of uncertainty and inconsistency leads to low happiness experienced in the present. However, the spillover effect of role involvement on wellbeing in the latter part of the model has not been confirmed. Perhaps because being involved is more of an experience of the present and does not involve the emotional experience of another domain at this time, the role involved in one domain acting on another domain should be “no happiness” or “no unhappiness,” not “happiness” or “unhappiness.”

## Practical Implications

First, we developed a Chinese version of the Family Centrality Questionnaire, which provides a scientific measurement tool for future research. Second, the study proves that Chinese employees’ role centrality, especially family centrality, has a significant impact on their work-family relationships and happiness. Therefore, the organizations should improve the awareness and ability of management to implement family friendly programs. Third, the results of the spillover effect show that a high level of family centrality can not only improve life wellbeing by increasing the level of family involvement but also decrease the level of life wellbeing because of negative spillover to work wellbeing. Thus, individuals should pay more attention to family and try their best to achieve work-family balance.

## Limitations and Future Directions

Although the present study helps improve the understanding of family centrality in China, it has several limitations that should be considered. First, the FCQ is a new scale that should continue to be tested, involving replications of these results and using the scale in different studies. Second, the data in this study were cross-sectional, and the sample was relatively single–mainly the grassroots employees of the factory. Further research must explore its nature longitudinally and across different groups as well as its relationship with other work and family outcomes. Third, this study only deals with the relationship between employees’ role centrality and wellbeing and does not explore the cross-over effects of family members’ role centrality on employee wellbeing. In the field of work and family research, cross-over and spillover effects are both very important. Future studies could examine how participants’ spouses’ or parents’ role centrality affects their work-family relationships and wellbeing.

## Conclusion

This study proved the independence of the concept of family centrality and validated the Chinese version of the family centrality scale. The result showed that the family centrality scale has good reliability and validity. At the same time, a preliminary application of the questionnaire showed that family centrality has a specific predictive effect on family related variables.

## Data Availability Statement

The raw data supporting the conclusions of this article will be made available by the authors, without undue reservation.

## Ethics Statement

The studies involving human participants were reviewed and approved by the Research Ethics Committee of the Institute of Psychology and Behavior, Henan University. The patients/participants provided their written informed consent to participate in this study.

## Author Contributions

XW and WW wrote the first draft of the manuscript. XW, WW, SW, GS, and YL performed the material preparation, data collection, data analysis, and commented on previous versions of the manuscript. All authors contributed to the article and approved the submitted version.

## Conflict of Interest

The authors declare that the research was conducted in the absence of any commercial or financial relationships that could be construed as a potential conflict of interest.

## Publisher’s Note

All claims expressed in this article are solely those of the authors and do not necessarily represent those of their affiliated organizations, or those of the publisher, the editors and the reviewers. Any product that may be evaluated in this article, or claim that may be made by its manufacturer, is not guaranteed or endorsed by the publisher.
